# Dynamics of vector competence for dengue virus type 2 in rural and urban populations of *Aedes albopictus*: implications for infectious disease control

**DOI:** 10.1186/s13071-025-06826-8

**Published:** 2025-06-01

**Authors:** Jehangir Khan, Muhammad Adil, Zhang Junyan, Dongjing Zhang, Yidong Deng, Zhiyue Lv, Tao Chen

**Affiliations:** 1 Hainan General Hospital, Hainan Affiliated Hospital of Hainan Medical University, Haikou, 570100 Hainan China; 2 Laboratory of Tropical Veterinary Medicine and Vector Biology, School of Life Sciences, Hainan University, Haikou, 570228 China; 3 Ministry of Education, Key Laboratory of Tropical Disease Control (Sun Yat-Sen University), Guangzhou, 510080 Guangdong China; 4 Hainan Provincial Bureau of Disease Prevention and Control, Haikou, 570100 China; 5 Zoology Department, Abdul Wali Khan University Mardan, Khyber Pakhtunkhwa, Mardan, 25000 Pakistan; 6 Pakistan Bureau of Statistics, Islamabad, 25000 Punjab Pakistan

**Keywords:** Vector competence, *Ae. albopictus*, DENV, Innate immunity, *Wolbachia*

## Abstract

**Background:**

Understanding the intrinsic factors that influence mosquito vector competence (VC) to pathogens is crucial for assessing the risk of disease transmission in both rural and urban environments. We assessed the VC of *Aedes albopictus* mosquitoes from urban (dengue-endemic) and rural (dengue-free) areas in Guangzhou, China, for dengue virus-2 (DENV-2), while also examining intrinsic factors such as *Wolbachia* and immune-related gene expression influencing VC.

**Methods:**

Adult females of rural, urban, and laboratory (control) populations of *Ae. albopictus* were orally exposed to a freshly prepared suspension of the DENV-2 New Guinea C strain (GenBank: AF038403.1), with a final titer of 1 × 10^7^ plaque-forming units (PFU)/ml, for a period of 60 min. Three different bioassays (B1–B3) were conducted on 60 mosquitoes per population: B1 at 7 days post-exposure (dpe) to assess viral infection in the mosquito midgut, and B2 and B3 at 14 dpe to evaluate viral dissemination in the carcass and transmission via saliva. The mosquito samples were processed for total RNA and DNA extraction. RNA was subsequently analyzed by quantitative reverse transcription polymerase chain reaction (qRT-PCR) to quantify viral load and measure the expression of immune-related genes, while DNA was assessed via quantitative PCR (qPCR) to determine *Wolbachia* density (*w*AlbA and *w*AlbB) and the rps6 gene.

**Results:**

At 7 dpe, virus proliferation in rural mosquitoes was similar to that in urban mosquitoes (*P* = 0.10). By 14 dpe, rural mosquitoes showed a significantly lower status of virus dissemination (*P* < 0.04) and transmission (*P* < 0.012). *Wolbachia* (−0.12 < *r* < −0.92) and immune effectors (−0.025 < *r* < −0.568) were negatively correlated with DENV in all mosquitoes, with more negative values indicating a stronger inverse relationship. The *w*AlbA and *w*AlbB strains exhibited similar densities across all the mosquito populations, with *w*AlbB revealing a slightly greater abundance in rural mosquitoes, although the difference was not significant. Elevated Relish 2 (*Rel2*), defensin A (*DefA*), and the signal transducers and activators of transcription (*STAT*) levels indicate activation of the Toll and JAK-STAT pathways, contributing to resistance against DENV replication and reduced VC in rural mosquitoes.

**Conclusions:**

This study indicates that rural *Ae. albopictus* mosquitoes may possess intrinsic barriers limiting their VC for DENV-2, offering valuable preliminary insights into VC across geographically distinct populations. However, further research across a broader range of urban and rural locations is needed to validate these findings and better understand the local factors influencing VC. Such insights are vital for public health, as they can help prioritize locations for dengue surveillance and effective vector control. Future studies should investigate the roles of intestinal microflora and immune pathways across diverse mosquito populations to better understand these dynamics and improve our knowledge of mosquito-borne disease transmission in different environments.

**Graphical Abstract:**

**Figure:**
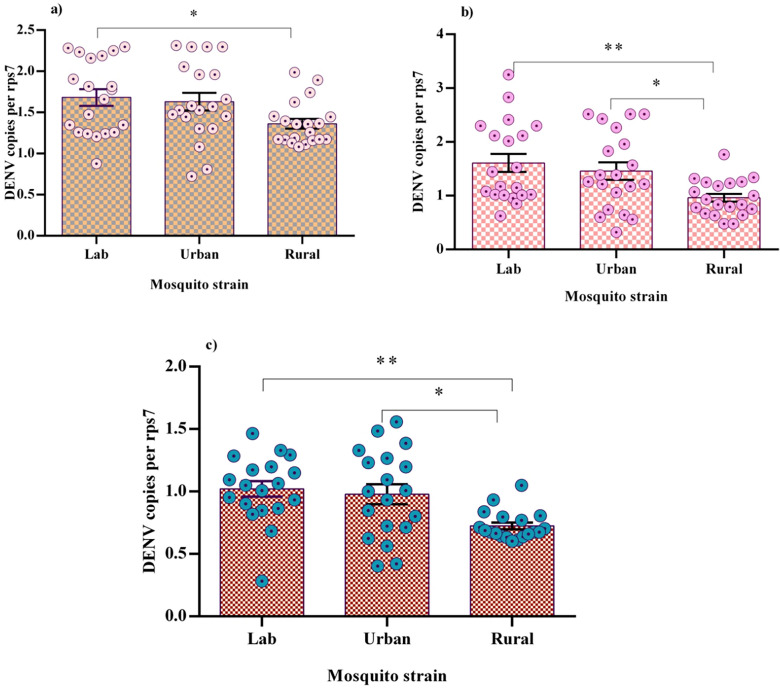

**Supplementary Information:**

The online version contains supplementary material available at 10.1186/s13071-025-06826-8.

## Background

Dengue fever, caused by multiple serotypes of dengue virus (DENV), is a significant public health concern affecting over 129 countries worldwide. In 2023, around five million cases and more than 5000 deaths were reported globally [1]. *Aedes albopictus*, a competent vector for at least 20 arboviruses, including DENV, has spread globally, thriving in diverse habitats from urban centers to rural areas and enhancing its vectorial capacity for DENV transmission [2–5].

The DENV transmission cycle begins when female mosquitoes ingest viral particles from infected vertebrates, including humans and non-human primates, eventually transmitting the virus to another host during subsequent blood meals [6]. Following an infectious blood meal, DENV levels peak in the mosquito midgut at between 7 and 10 days, in the salivary glands between 10 and 17 days (the extrinsic incubation period [EIP], during which the mosquito becomes capable of transmission), and in the head after 14 days [7, 8]. Vector competence (VC), which refers to the mosquito’s intrinsic ability to acquire, maintain, and transmit the virus, plays a critical role in the transmission cycle. To complete the EIP, the virus must overcome three key barriers—the midgut infection barrier (MIB), midgut escape barrier (MEB), and salivary gland barrier (SGB)—all of which influence VC [7]. The MIB prevents infection or replication in the midgut, while the MEB restricts the virus's ability to escape the midgut, and the SGB hinders the virus’s passage into the salivary glands [7, 8].

The VC of a mosquito is influenced both by extrinsic factors such as habitat ecology and internal factors including virus strain, genetics, immunity, and the intestinal microbiota [6, 9–11]. The vectorial capacity of a mosquito population to transmit a pathogen is influenced by various factors, including VC, which encompasses infection, dissemination, and transmission rates, as well as the mosquito’s lifespan and biting frequency, all of which together determine transmission dynamics [12]. Notable heterogeneity in VC for different DENV serotypes has been reported among *Ae. albopictus* populations from diverse geographical locations and ecological settings [7, 12–16].

*Wolbachia*, a genus of symbiotic bacteria, plays a crucial role in modulating VC by inhibiting pathogen transmission in mosquitoes. *Aedes albopictus* naturally harbors superinfections of the *Wolbachia* strain *w*AlbA and *w*AlbB. However, due to inadequate density, *Wolbachia* fails to induce strong DENV resistance, as higher density correlates negatively with DENV load [17]. *Wolbachia* trans-infection in *Ae. aegypti*, a non-native host, effectively blocks dengue and Zika virus transmission [18, 19], contributing to successful disease control. *Wolbachia*-mediated inhibition of DENV occurs through mechanisms such as immune activation, resource competition, and alterations in the mosquito’s intracellular environment [10]. Key immune pathways involved in this process include the Janus kinase/signal transducer and activator of transcription (JAK-STAT), Toll, and immune deficiency (Imd) pathways [20]. Relish 1 (*Rel1*), *Rel2*, cecropin A (*CecA*), defensin (*DefA*), and *STAT* are the key immune factors that show significant effectiveness against viral replication in mosquitoes [21–24].

VC is also influenced by the larval environment across various habitats [25–27]. Larvae acquire microbes and immune-modulating factors from aquatic larval habitat, which can carry over into adulthood and affect the mosquito’s ability to replicate and transmit the virus [25–31]. Local environmental conditions, including urban and rural settings, play a key role in determining the VC of mosquito populations [7, 12, 13, 32] and influencing flavivirus transmission dynamics [6, 33–39]. For instance, urban *Ae. aegypti* in Ghana exhibited higher VC for DENV than rural populations [40]. More recently, *Ae. albopictus* mosquitoes from high-dengue-incidence areas exhibited greater DENV-2 VC than those from low-incidence areas in Yunnan province, China [6], corroborating an observation from another study [33].

Guangzhou, which has been affected by dengue since it was first reported in 1978 [41], accounted for 80% of all nationally reported cases in China in 2014 [41]. Despite *Ae. albopictus* being a major vector of DENV, dengue outbreaks and autochthonous transmission predominantly occurred in urban areas of Guangzhou until 2014 [5, 41–43]. Factors such as urbanization, favorable climate conditions, and increased human mobility have driven the expansion of *Ae. albopictus*, contributing to sustained DENV outbreaks in urban areas [43]. Given that these factors are less pronounced in rural areas, where dengue outbreaks have historically been absent, it is crucial to investigate whether differences in the habitat of origin (urban vs. rural) influence the VC of *Ae. albopictus* populations. This raises important questions about the relative susceptibility of urban versus rural *Ae. albopictus* populations to DENV and whether rural mosquitoes are less efficient vectors of dengue.

This study primarily aimed to compare the VC of *Ae. albopictus* mosquitoes for DENV-2 by assessing infection, dissemination, and transmission levels between urban (dengue-endemic) and rural (dengue-free) populations in Guangzhou. To provide a deeper understanding of the biological mechanisms underlying VC, the study also evaluated intrinsic factors, such as the density of natural *Wolbachia* infections and the expression of immune-related genes. By integrating these analyses, this study provides critical insights into the interplay of intrinsic factors and population-level differences in VC, offering a comprehensive understanding of DENV-2 transmission potential in distinct mosquito populations. These findings aim to inform and refine strategies for vector control and dengue prevention.

## Methods

### Description of the sampling area

Guangzhou (GZ 112°57 E to 114°3 E and 22°26 N to 23°56 N) is a metropolitan city (7434 km^2^ with approximately 15.31 million people) and the capital of Guangdong Province [41], further described elsewhere [42]. Located on the northern edge of the Pearl River Delta, this international business center, renowned for its numerous popular tourist attractions, features an oceanic subtropical monsoon climate. The annual mean temperature is 20–22 °C, the average relative humidity is 77%, and the annual rainfall is about 1720 mm. This favorable climate, combined with abundant breeding habitats, provides a suitable environment for *Ae. albopictus*, a competent vector for arboviruses, including dengue and Zika viruses, in the region. Many large dengue outbreaks have been recorded in Guangzhou, with urban areas being particularly impacted [41–43].

### Mosquito sampling and rearing

#### Sampling sites

Mosquito sampling was conducted during the dengue transmission season (July to September 2019) in both dengue-free rural communities (e.g., Meilin Lake Community, MLC) and urban dengue hotspots (Yuexiu District). MLC features abundant vegetation, scattered water bodies, and diverse wildlife from complex ecosystems. These environmental characteristics contribute to habitat suitability and mosquito breeding. While no dengue cases have been previously reported, the influx of national and international travelers, including both permanent and temporary residents, from dengue-endemic regions during the summer may significantly increase the risk of dengue outbreaks. It was also the proposed target area for field trials of *Wolbachia* deployment for mosquito population suppression [44]. Yuexiu District, an urban hotspot covering approximately 31.29 km^2^ with a population density of around 34,000 people/km^2^ (based on a population of 1.15 million), is characterized by a mix of high-rise residential buildings, commercial structures, and older, densely packed housing, and experiences frequent dengue outbreaks and high mosquito densities [42].

#### Sampling methods

Sampling was conducted using ovitraps placed at 15–20 sampling points in each area for ova/larvae collection, as described in our previous study [44]. This ovitrap is a small, black cup filled with water up to one third of its volume. Inside, there is a piece of egg collection paper that is specifically designed to improve mosquito oviposition. The collected eggs and hatching larvae were subsequently brought to the laboratory (details given below) for rearing.

### Laboratory rearing

The collected larvae were reared under standardized conditions in an insectary (BSL-2) at the Zhongshan School of Medicine, Sun Yat-sen University (SYSU) [45], and colonies from rural and urban areas were established. After emergence, about 120 adult mosquitoes (males/females = 1:1) were randomly selected from the samples from each site and mixed together to mate. The adult mosquitoes were provided with animal blood using a Hemotek membrane feeding system (Hemotek Ltd., UK), a commercially available device designed for artificial blood feeding of mosquitoes [46], and the eggs were collected after 3–4 days. To ensure a sufficient mosquito number, adult *Ae. albopictus* second-generation (G2) mosquitoes were used in our study. Adult mosquitoes were maintained in cages at 27 ± 1 °C and 80% relative humidity under a 12 h light/12 h dark cycle and were fed a 10% sucrose solution [45].

### Control strain

In this investigation, we used three mosquito populations: rural, urban, and laboratory. The laboratory strain was originally sourced from the same urban location, but it has been maintained in the lab for more than 2 years, earning it the “lab strain” designation. Our previous experiments demonstrated the susceptibility of this laboratory strain to DENV-2 infection. Consequently, this mosquito was used as a control (standard) to facilitate the differentiation in VC between rural and urban mosquito populations. The laboratory strain, characterized by higher susceptibility, indicates an intrinsic sensitivity that may not be present in wild populations but is a useful model for understanding the potential for DENV transmission under controlled conditions.

### Screening for natural DENV infection

Prior to conducting laboratory infections, we screened a subset of larvae using quantitative reverse transcription polymerase chain reaction (qRT-PCR) to ensure that there was no pre-existing DENV infection [12]. This step was important for ruling out the possibility of vertical transmission, which, while rare, can occur at low frequencies in dengue-endemic areas. We tested (*n* = 100) larvae, randomly selected from each population, to verify that the adult females derived from these larvae were free of natural DENV infection. Total RNA was extracted from pools of 10 larvae each, resulting in 10 pools per population [44]. By confirming the absence of the virus before proceeding with our experiments, we ensured that any DENV detected post-exposure was solely a result of our laboratory procedures.

### Viral strain and mosquito infection

The DENV-2 virus (New Guinea C strain, GenBank: AF038403.1) was obtained from the Center for Disease Control and Prevention (CDC) in Guangzhou, Guangdong Province. DENV was inoculated and passaged in *Ae. albopictus* C6/36 cells (six-well plate) for 5 days at 35 °C (above the standard 28–30 °C range, selected to enhance DENV-2 replication efficiency) and 5% CO_2_, following our previous protocols [5, 44]. Afterwards, the virus was extracted and titrated immediately via plaque assay. Briefly, a medium containing 2% methylcellulose dissolved in 100 ml of Dulbecco’s modified Eagle medium (DMEM) supplemented with 2% fetal bovine serum (FBS) was used to confine virus proliferation to distinct plaques [44]. After 5 days of virus inoculation, the cells were fixed with 4% paraformaldehyde and stained with hematoxylin. Plaques were counted using a microscope (Leica DM750) to determine the virus titer. The titer of the harvested DENV-2 suspension was determined to be 2.1 × 10^7^ plaque-forming units (PFU)/ml (range 1.8–2.3 × 10^7^ PFU/ml across all preparations) [5]. The virus suspensions were then aliquoted and stored at −80 °C until use. Before the infectious blood meal, 3- to 4-day-old female mosquitoes were starved for 24 h. To prepare the infectious blood meal, the titrated DENV-2 suspension was mixed with an equal volume of anticoagulant blood, resulting in a final virus concentration of approximately 1 × 10^7^ PFU/ml [44]. About 100 female adult mosquitoes (from each population) were allowed to feed for around 1 h on the infectious blood meal [44]. The fully engorged mosquitoes were subsequently isolated and transferred into cardboard containers. They were maintained for 14 days, the period required to complete the EIP, at 27 ± 1 °C and 80% relative humidity, with 12 h light/dark cycles, and were fed a 10% sucrose solution [44, 45].

### Mosquito dissection and tissue isolation for multiple bioassays

We performed three bioassays (B1–B3) on a total of 60 female mosquitoes per population, randomly collected from each group after exposure to an infectious blood meal containing DENV-2. In this study, a bioassay refers to a controlled experiment designed to assess the presence and quantity of DENV-2 in specific mosquito tissues (midgut, carcass, and saliva) at defined time points post-exposure, to determine infection, dissemination, and transmission levels. Prior to the dissection process, mosquitoes were anesthetized with CO_2_, washed with 70% ethanol, and rinsed with sterile water. B1 was conducted after 7 days post-exposure (dpe), during which DENV infection levels were assessed in the midguts of 20 mosquitoes per population [24]. B2 and B3 were conducted at 14 dpe. For both assays, 40 mosquitoes were sampled from each population. In B2, dissemination levels were assessed by examining the carcasses (mosquito bodies excluding the heads). In B3, transmission levels were evaluated by analyzing the saliva from the same mosquito, following the methodology described by Goncalves et al. [12]. Saliva collection was performed through forced salivation tests, as described previously [8, 47]. In short, mosquitoes were starved for 24 h before undergoing forced salivation. Following CO_2_ exposure for immobilization, the mosquitoes were removed from CO_2_, and their legs and wings were dissected to facilitate the procedure. The proboscis was then inserted into a 10 μl pipette tip containing 6 μl of FBS, where it remained for 60 min at room temperature to expectorate saliva [5]. Given the small sample size, saliva from two mosquitoes was pooled into 20 replicates, and the carcasses were similarly pooled for consistency, as per established methods [8, 22]. All the tissues were dissected in sterile PBS, transferred separately to microcentrifuge tubes containing 100 µl of TRIzol, and homogenized using a homogenizer.

Total RNA and/or DNA were extracted from samples using the procedure outlined below. RNA was used to measure viral load (via DENV-2 NS5 copies) and immune gene expression through qRT-PCR, while DNA was used to assess (via quantitative PCR [qPCR]) *Wolbachia* density (*w*AlbA and *w*AlbB) and the *rps6* gene [13, 22]. Infection, dissemination, and transmission were evaluated by detecting DENV in the midgut at 7 days dpe, the carcass at 14 dpe, and the saliva at 14 dpe using qRT-PCR to quantify DENV RNA copies in each tissue [16, 47]. This approach focused on measuring DENV RNA replication rather than determining the proportions of infected mosquitoes.

### Extraction and quantification of total RNA

Total RNA was extracted using TRIzol reagent (Invitrogen, Carlsbad, CA) and complementary DNA (cDNA) was synthesized using the PrimeScript RT Reagent Kit (Takara Bio, Otsu Shiga, Japan) following the manufacturer’s instructions. The number of viral RNA copies was measured using qPCR on a LightCycler 96 Detection System from Roche, employing TB Green Premix Ex Taq II (Tri RNase H Plus) from Takara under the following conditions: initial denaturation at 95 °C for 30 s, 40 cycles of denaturation at 90 °C for 5 s and annealing/extension at 60 °C for 30 s, concluding with a melting curve analysis. The *rps6* gene (*Ae. albopictus*) was used as an internal control for normalization of RNA levels according to previous protocols [5, 13]. To quantify the virus, the DENV NS5 and mosquito *rps6* genes were individually cloned and inserted into the pMD-18 T vector from Takara, following the method outlined previously [5]. Standard curves were generated using serial dilutions (from 10^1^ to 10^8^ copies/reaction) of these recombinant plasmids [8]. Moreover, immune-related gene quantification in dengue-infected mosquitoes used standard curves from recombinant plasmids with *Rel1*, *Rel2*, *CecA*, *STAT*, and *DefA* gene fragments [5, 13, 22, 48]. The primers (Supplementary Text S1) for DENV, rps6, *Wolbachia*, and immune-related genes, with a slight modification in the thermal profiles, were adapted from prior research [5, 8, 13, 21]. The expression levels of innate immune genes were tested by qRT-PCR and determined by using the 2^−△△CT^ calculation method as described previously [5, 8, 49].

### *Wolbachia* quantitative PCR

Genomic DNA extraction was carried out using the Takara DNA extraction kit according to the manufacturer’s protocols. Quantitative PCR, following the protocol described previously [8], was used to quantify the *Wolbachia* genome copies in mosquito samples. Standard plasmids containing target fragments of *Wolbachia* strain A (*w*AlbA) and strain B (*w*AlbB) were used to quantify *Wolbachia* densities (via qPCR) in mosquitoes and normalized with the housekeeping gene *rps6* [13].

### Statistical analysis

All the data were statistically analyzed using SPSS, R, and GraphPad Prism (version 5) to determine differences in replication levels of DENV-2, *Wolbachia* surface protein, and innate immune genes between different mosquito populations. Additionally, we conducted analysis of variance (ANOVA) and multiple-comparison tests (least significant difference [LSD]). The Spearman correlation was also calculated to determine the associations between infection, dissemination, transmission, and *Wolbachia* and immune-related genes. Moreover, we constructed box-and-whisker plots and scatter plots using ggplot2 in R software. Values of *P* < 0.05 were considered statistically significant.

## Results

### Differences in infection, dissemination, and transmission of DENV across mosquito populations

**Fig. 1:**
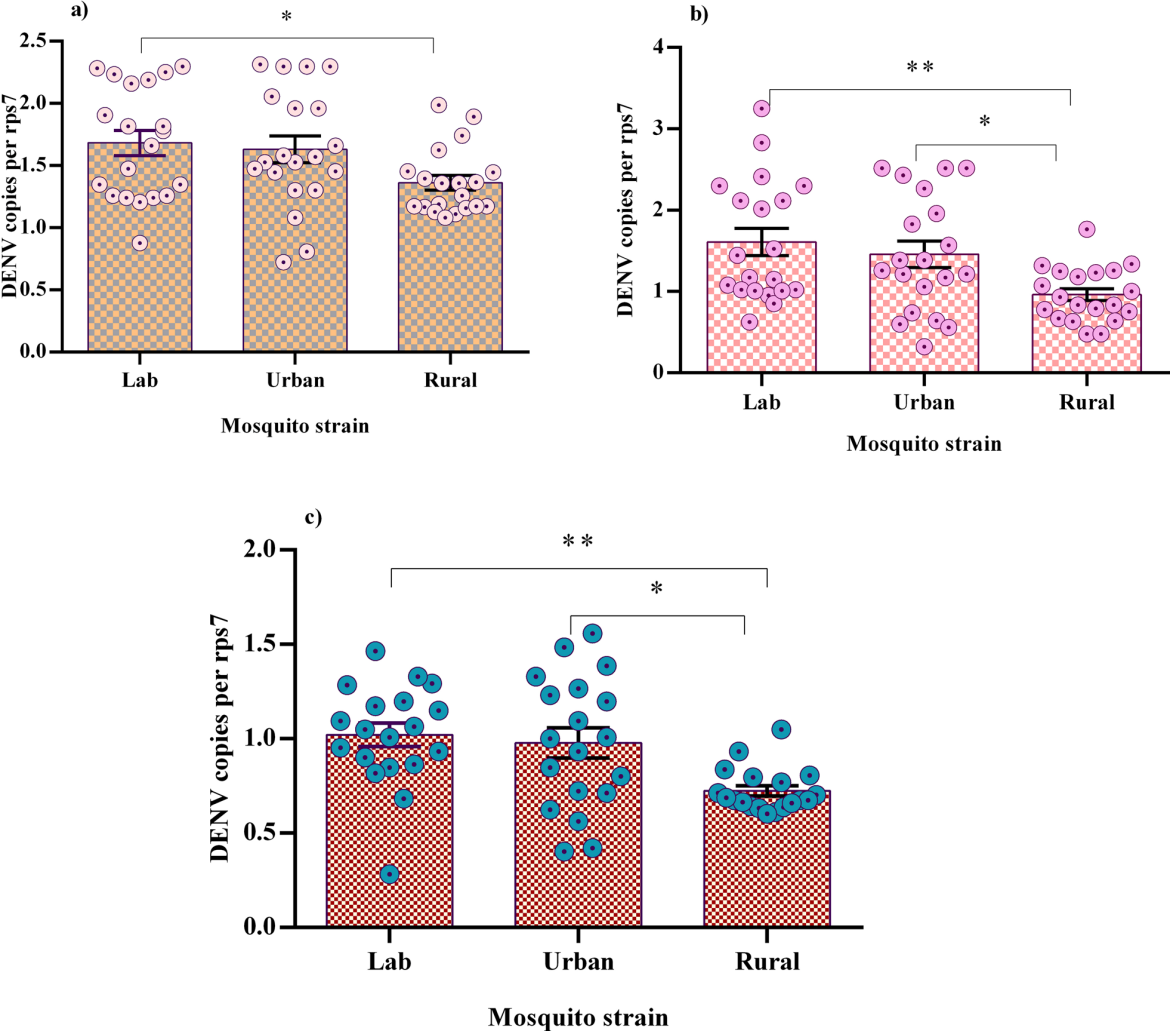
DENV-2 RNA replication in three mosquito populations, measured as DENV copies per PFU, showing midgut at 7 dpe (**a**), carcass at 14 dpe (**b**), and saliva at 14 dpe (**c**). Horizontal lines represent the mean with standard error of the mean (SEM). Significant differences are marked by asterisks (*). *Indicates *P* < 0.05; **indicates *P* < 0.01

Prior to in vitro laboratory infection, qRT-PCR screening of field-collected larvae confirmed the absence of natural DENV infection. At 7 dpe, qRT-PCR analysis revealed that DENV-2 genome copies were comparable between rural and urban mosquitoes (*P* = 0.10), although rural mosquitoes exhibited significantly reduced replication compared to the sensitive lab strain (*P* = 0.04). At 14 dpe, rural mosquitoes showed reduced dissemination (*P* = 0.04) and transmission (*P* = 0.0124) capacity compared to both urban and lab groups (Fig. 1). Although DENV replication surged in the carcasses of all populations, viral RNA levels in saliva remained low (Fig. 1), suggesting restricted replication in this tissue. Moreover, no significant differences were observed in DENV genome copies during infection (7 dpe) and dissemination (14 dpe) phases between urban and lab mosquitoes (*P* > 0.05), indicating consistent viral replication patterns.

### Immune gene expression patterns and their correlation with DENV load across mosquito populations

#### Gene expression patterns

**Fig. 2:**
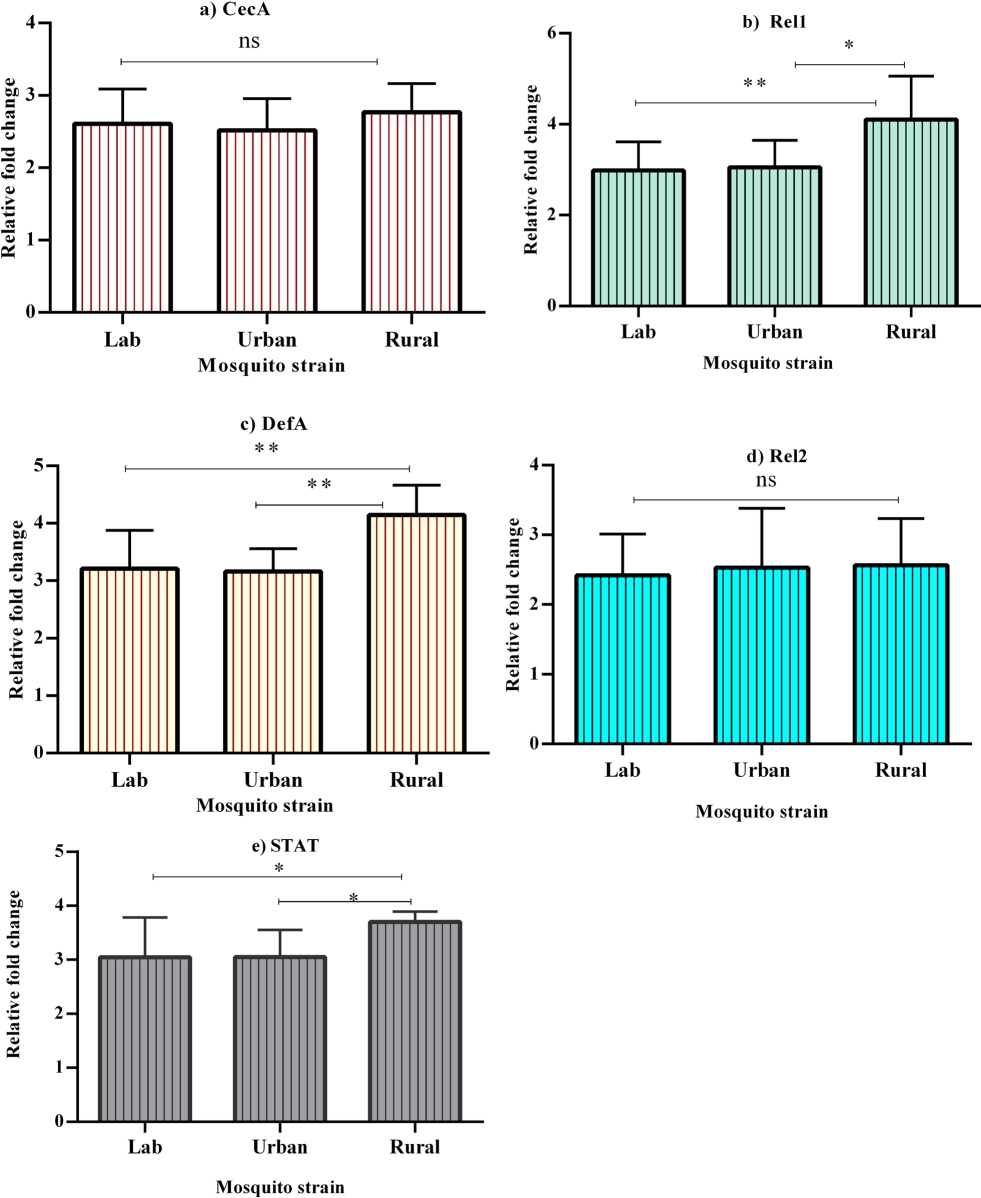
Relative expression of basic immune genes in three mosquito populations. Bars indicate means with standard error of the mean (SEM). Significant differences are indicated by asterisks (*). *Indicates *P* < 0.05; **indicates *P* < 0.01

The relative expression levels of key immune genes (*CecA*, *Rel1*, *DefA*, *Rel2*, and *STAT*) were analyzed across the lab, urban, and rural mosquito populations (Fig. 2). *CecA* and *Rel2* showed no significant differences among the populations, indicating consistent expression levels irrespective of environmental or population factors. In contrast, *DefA*, *Rel1*, and *STAT* expression levels were significantly higher, especially in the rural mosquitoes, compared to both the lab (*P* < 0.01) and urban (*P* < 0.05) populations (Fig. 2), with no significant difference between the latter two groups. Post hoc LSD tests (supplementary File Table 1) further showed that *Rel1* expression was significantly higher in rural mosquitoes than in lab (*P* = 0.002) and urban (*P* = 0.004) populations. *DefA* expression differed across all pair-wise comparisons: rural vs. urban (*P* < 0.001), rural vs. lab (*P* = 0.001), and lab vs. urban (*P* < 0.001). Similarly, *STAT* expression was significantly elevated in rural mosquitoes compared to both lab (*P* = 0.010) and urban (*P* = 0.010) populations, with significant differences also observed between the lab and urban populations (*P* = 0.013).

**Table 1:** ANOVA and LSD tests for mean difference analysis of *Wolbachia* and DENV replication across mosquito populations

Variables		Sum of squares	*df*	Mean square	*F*	Sig
Transmission	Between groups	0.972	2	0.486	6.991	0.002
	Within groups	3.755	54	0.070		
	Total	4.728	56			
Dissemination	Between groups	4.965	2	2.483	6.112	0.004
	Within groups	21.933	54	0.406		
	Total	26.899	56			
*Wolbachia*	Between groups	0.225	2	0.113	2.858	0.066
	Within groups	2.128	54	0.039		
	Total	2.353	56			
Infection	Between groups	0.927	2	0.463	2.764	0.072
	Within groups	9.053	54	0.168		
	Total	9.980	56			

Multiple comparisons (LSD) for dissemination and transmission across various mosquito populations							
Dependent variable			Mean difference (*I*–*J*)	Std. error	Sig	95% confidence interval	
						Lower bound	Upper bound
Transmission	Lab	Urban	0.04251	0.08556	0.621	−0.1290	0.2140
		Rural	0.29586*	0.08556	0.001	0.1243	0.4674
	Urban	Lab	−0.04251	0.08556	0.621	−0.2140	0.1290
		Rural	0.25336*	0.08556	0.005	0.0818	0.4249
	Rural	Lab	−0.29586*	0.08556	0.001	−0.4674	−0.1243
		Urban	−0.25336*	0.08556	0.005	−0.4249	−0.0818
Dissemination	Lab	Urban	0.17357	0.20677	0.405	−0.2410	0.5881
		Rural	0.69456*	0.20677	0.001	0.2800	1.1091
	Urban	Lab	−0.17357	0.20677	0.405	−0.5881	0.2410
		Rural	0.52100*	0.20677	0.015	0.1064	0.9356
	Rural	Lab	−0.69456*	0.20677	0.001	−1.1091	−0.2800
		Urban	−0.52100*	0.20677	0.015	−0.9356	−0.1064

*The mean difference is significant at the 0.05 level

### Correlation analysis of DENV-2 load and immune gene expression

**Fig. 3:**
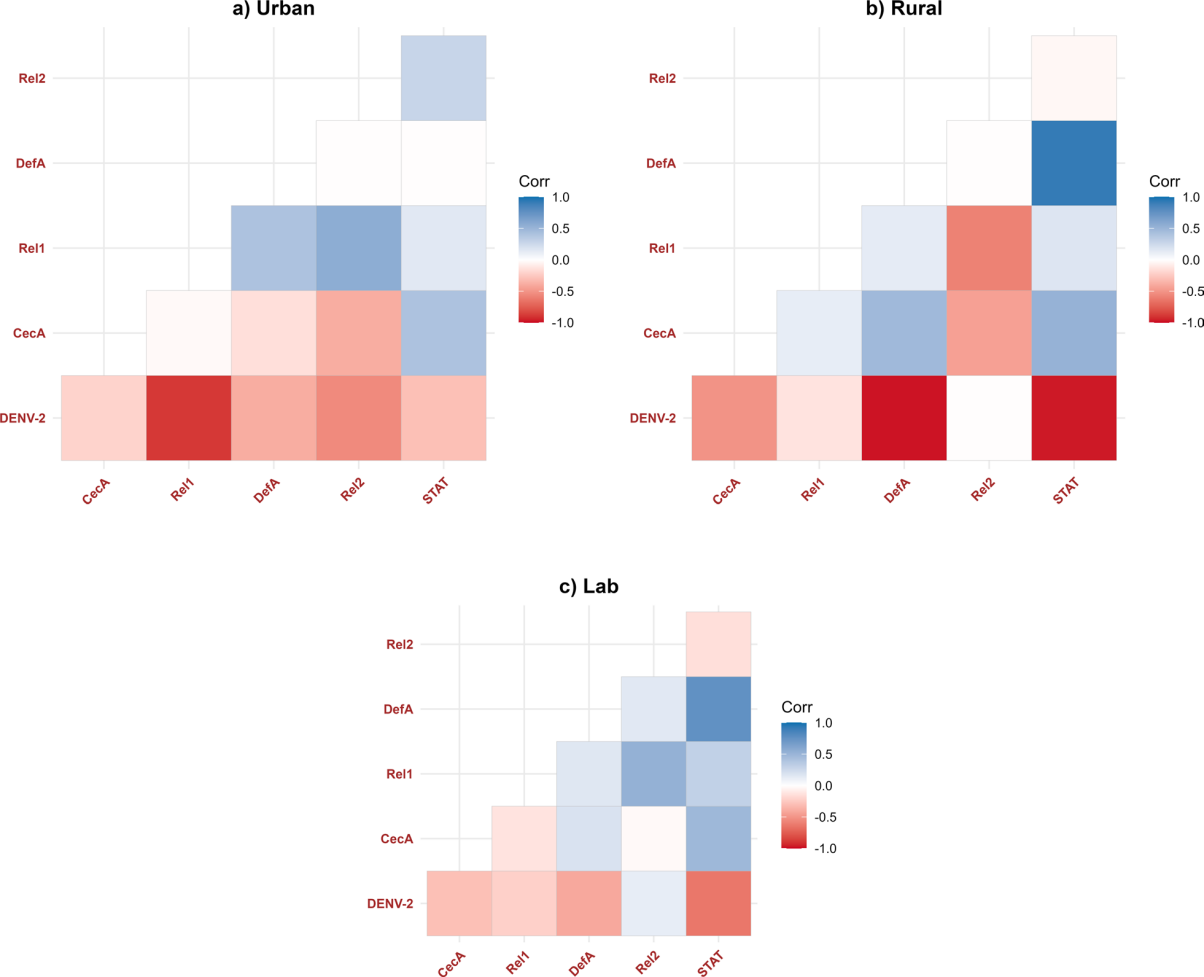
Correlation heat map illustrating the relationship between DENV loads and immune-related gene expression across mosquito populations: urban (**a**), rural (**b**), and lab (**c**). The color intensity represents the strength and direction of the correlation. Shades of red indicate negative correlations, with darker red representing stronger negative associations (ranging from 0 to −1). Shades of blue indicate positive correlations, with darker blue representing stronger positive associations (ranging from 0 to +1). Lighter colors (closer to white) reflect weaker correlations closer to zero

The correlation analysis between DENV-2 loads and the immune-related genes (*CecA*, *Rel1*, *DefA*, *Rel2*, and *STAT*) revealed population-specific patterns (Fig. 3). Strong negative correlations were consistently observed for *Rel1*, *DefA*, and *STAT*, with *STAT* emerging as a key immune regulator, particularly in the rural population. In rural mosquitoes, significantly negative correlations were observed for *DefA* (*P* = 0.030) and *STAT *(*P* = 0.047). In urban mosquitoes, *Rel1* (*P* = 0.06), *DefA* (*P* = 0.261), and *STAT *(*P* = 0.402) showed negative correlations with DENV-2 loads, though not statistically significant. In lab-reared mosquitoes, *STAT* displayed a strong negative correlation (*P* = 0.056). Weak or moderate correlations were observed for *Rel1* and *CecA* in certain populations. However, *Rel2* demonstrated weak positive correlations in lab populations.

### *Wolbachia* density patterns and DENV replication dynamics across mosquito populations

**Fig. 4:**
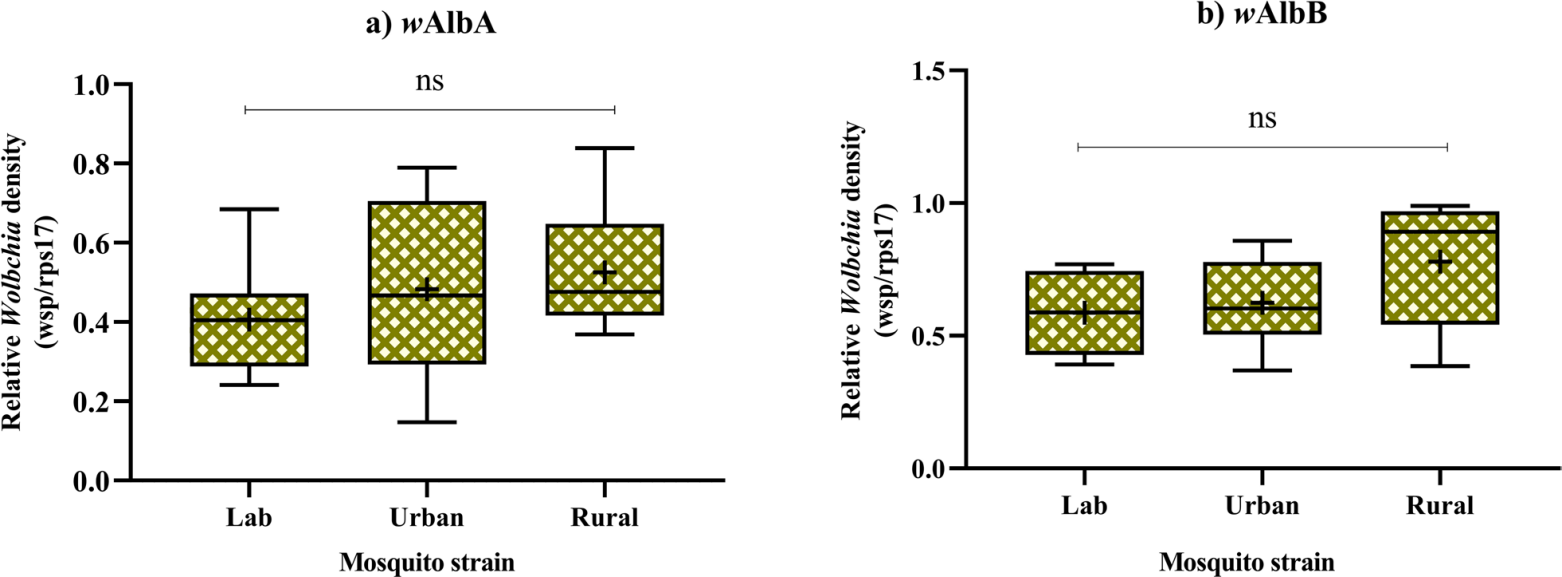
Relative density of *Wolbachia* (*w*AlbA and *w*AlbB) in female *Ae. albopictus* in three mosquito populations. Bars indicate the mean with standard error of the mean (SEM)

**Fig. 5:**
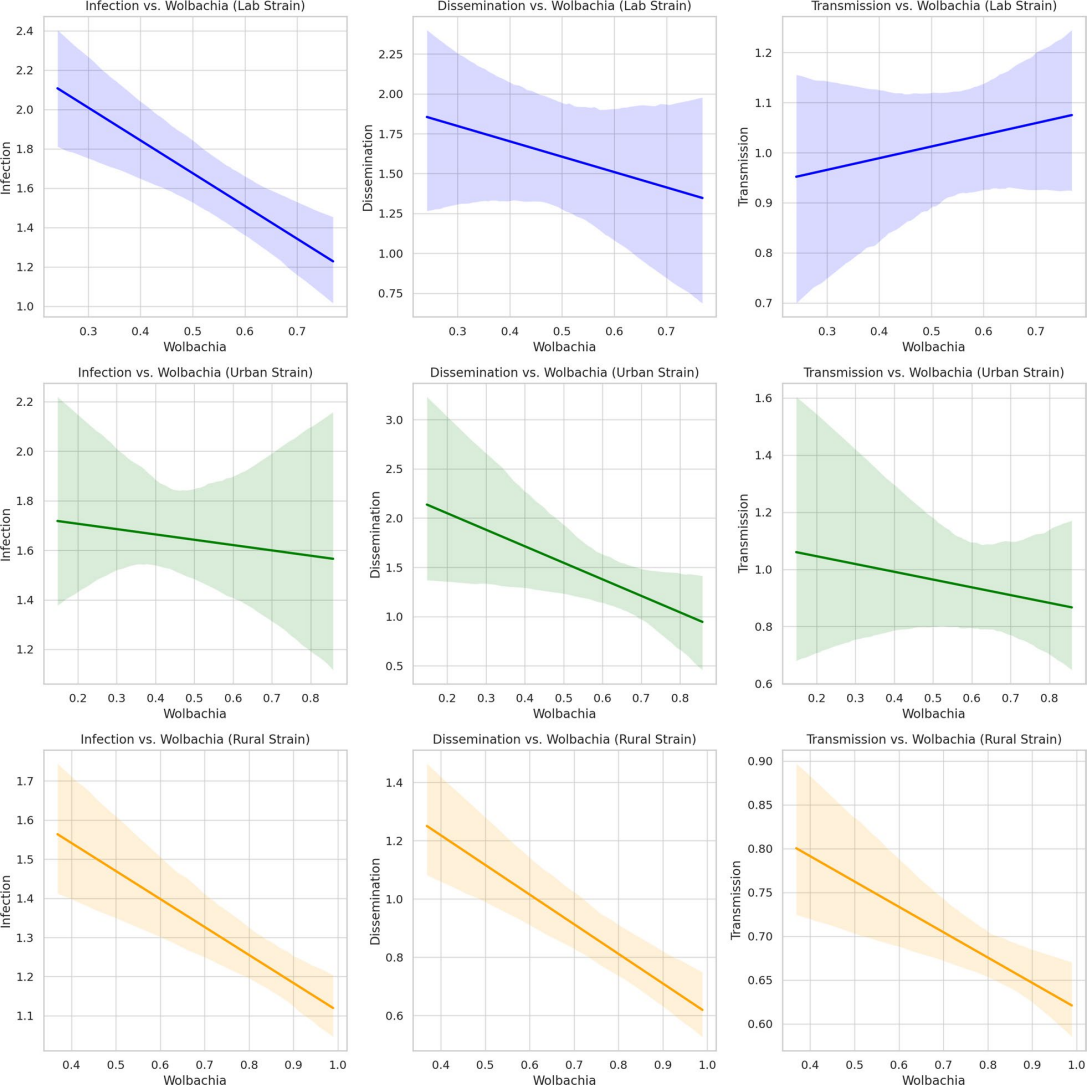
Relationship between *Wolbachia* density and infection, dissemination, and transmission levels across mosquito strains. The shaded regions represent the variability in the data; the color scheme distinguishes between mosquito strains: lab (blue), urban (green), and rural (orange). The shaded regions represent the variability in the data, illustrating the range of values observed. The solid trend lines show the direction and strength of the correlation, with steeper slopes indicating stronger negative correlations

Given that *Wolbachia*-mediated pathogen blockade in *Ae. albopictus* is correlated with *Wolbachia* density, which varies across several key somatic tissues [17], we quantified *Wolbachia* density in individual female mosquitoes (Fig. 4) to investigate its potential influence on DENV replication. While overall *Wolbachia* density did not differ significantly among populations, the density of *w*AlbB consistently exceeded that of *w*AlbA across all groups (Fig. 4). Rural mosquitoes showed slightly higher *w*AlbB abundance than urban and lab populations, although this difference was not statistically significant. A negative correlation between *Wolbachia* density and DENV replication was observed across all populations, with statistical significance achieved only in rural mosquitoes (Fig. 5 and Table 1). This finding corresponds with the reduced dissemination and transmission levels observed in rural mosquitoes compared to urban and lab populations (Fig. 1). Interestingly, the correlation between *Wolbachia* density and DENV RNA in the saliva of lab mosquitoes was weak, suggesting that the *Wolbachia*–DENV-2 relationship may not be linear in certain mosquito tissues, as also reported by Amuzu and McGraw [50].

## Discussion

Understanding the factors that shape VC in *Ae. albopictus* is critical for addressing dengue outbreaks, particularly in areas with contrasting levels of DENV transmission, such as urban and rural environments. Our study highlights the significant differences in VC between rural (dengue-free) and urban (dengue-endemic) *Ae. albopictus* populations, with rural mosquitoes demonstrating reduced DENV replication and transmission. These findings shed light on how habitat-specific selective pressures can influence the intrinsic traits of mosquito populations, offering valuable insights for designing targeted interventions to disrupt the dengue transmission cycle. Rural populations, isolated from endemic DENV circulation, appear to have retained the intrinsic barriers, such as MEB, SGB, or mechanisms restricting viral replication. These physiological barriers likely account for the significantly reduced dissemination and transmission observed in rural mosquitoes. Similar habitat-driven differences in VC have been reported in other studies [12, 33], underscoring the complex interplay between environmental factors and mosquito–virus interactions. The refractoriness observed in rural mosquitoes highlights the potential of habitat-adapted mosquito traits for vector control. Population replacement strategies could exploit these traits to introduce mosquito populations less susceptible to arbovirus. For example, *Wolbachia*-based approaches that rely on introducing mosquitoes with reduced VC may benefit from the identification of refractory traits unique to rural mosquito populations. However, further research is needed to validate these traits under field conditions and evaluate their feasibility for scalable application [44].

In contrast, urban mosquitoes exposed to DENV circulation and anthropogenic pressures, such as insecticide use, may experience selective pressures that could alter their intrinsic traits related to VC and increasing susceptibility [13, 37, 51, 52]. This ecological divergence between urban and rural populations underscores the impact of evolutionary dynamics on mosquito–virus interactions [33, 37, 51]. The lack of significant differences in DENV replication between urban and laboratory populations further suggests that environmental and habitat-specific factors predominantly contribute to the distinct refractoriness observed in rural mosquitoes.

Our findings, demonstrating increased VC in mosquitoes from dengue-endemic areas, align with previous studies that report heightened susceptibility in mosquito population from dengue-endemic areas [6, 7, 33, 37]. In contrast, our results differ from earlier reports indicating lower viral loads in *Ae. albopictus* from endemic areas in China [51]. While some international studies corroborate our findings [37, 40, 53–55], others present contrasting results [56]. Similarly, some pioneering research has documented increased susceptibility to Zika [15] and DENV [57] in urban *Ae. albopictus* in Guangzhou/Guangdong, echoing our findings and underscoring co-circulation concerns in epidemiological contexts. In contrast, we observed a lower DENV load compared to previous studies [13, 16, 57], despite using a comparatively higher virus titer. Such discrepancies likely arise from differences in ecological environments, mosquito strains, viral genotypes, or methodological approaches. These variations underscore the importance of standardizing research protocols across regions and integrating ecological and temporal variability into experimental designs to better understand VC dynamics.

The role of *Wolbachia* in mosquito–virus interaction has been well documented, with density- and strain-specific efforts significantly influencing VC [6, 22, 23, 58, 59]. In our study, rural populations exhibited slightly higher *w*AlbB abundance than urban and lab mosquitoes, which may partially explain their reduced VC, an observation similar to findings in previous studies [3, 13, 47]. The *w*AlbB is known to confer density-dependent antiviral effects by enhancing innate immune responses and limiting viral replication, particularly in critical tissues such as the salivary glands, which directly determine transmission potential [17, 19, 22, 23, 58, 61]. This effect likely explains the reduced DENV RNA level in salivary glands in all mosquito populations, particularly in the rural population, and supports the hypothesis that *Wolbachia* inhibits viral progression by interfering with tissue-specific dissemination [14, 17, 22, 23, 62]. These findings are consistent with those of previous studies [12, 15, 16, 22, 23, 37, 40, 57, 63]. Similarly, a previous study revealed that *Wolbachia* in *Ae. albopictus* does not impede viral replication but significantly reduces the viral density in the salivary glands, limiting DENV transmission [14]. Together, these observations suggest the activation of distinct *Wolbachia* density- or time-dependent virus-inhibiting mechanisms, potentially leading to more pronounced inhibition of subsequent virus proliferation [17, 22]. Interestingly, susceptibility levels in the same *Aedes* species vary over different time periods within the same location [12], indicating that temporal/demographic factors influence VC [2, 37]. Furthermore, recent findings [6] regarding the intestinal bacterial fauna of *Ae. albopictus* in the same geographical area led us to hypothesize that the combined effects of *Wolbachia* and other symbiotic midgut bacteria may synergistically contribute to inhibiting DENV replication in mosquitoes from dengue-free areas. The microbial composition can vary significantly even among microclimates within a single location [60], reflecting the impact of habitat variability, a finding that is consistent with those observed in other studies [2, 6, 64]. While microbial communities may influence VC, regional variations in *Ae. albopictus* competence for DENV transmission likely result from multiple factors, including mosquito genotypes and environmental conditions [63, 65]. Diverse larval habitats shape adult mosquito microbial communities (acquired from both mothers and the environment), which vary notably even over short distances [6, 66]. Intestinal bacteria activate immune pathways, increasing the production of antipathogenic compounds to eliminate pathogens [67–70]. Such findings highlight the importance of understanding how microbial ecosystems influence mosquito immunity and susceptibility to arboviruses. We hypothesize that coevolution with microbial communities and exposure to diverse DENV genotypes have shaped the immune repertoires of mosquito populations. This could result in distinct gut microbiota that modulate traits such as VC, particularly in rural mosquitoes. Understanding this interplay among microbiota, mosquito physiology, and ecological factors is vital for designing effective vector control strategies tailored to specific environments.

Mosquito antiviral defenses are mediated primarily by innate immune pathways such as the JAK-STAT, Toll, and Imd [5, 8, 71–73]. The commensal microbiota, including *Wolbachia*, shapes basal immunity, as its removal enhances susceptibility to pathogens [22, 74, 75]. Habitat-driven differences likely influence immune gene expression, potentially in a *Wolbachia* density-dependent manner, as suggested by others [17, 19]. Our study demonstrated slightly elevated expression levels of immune-related genes and *Wolbachia* density compared to other studies in the same area [13], suggesting that ecological and microbial factors contribute to observed differences in VC, a pattern similarly noted in previous studies [22, 76]. Among the immune genes examined, *DefA*, *Rel2*, and *STAT* exhibited significant differential expression, with robust activity in rural mosquitoes. This highlights the enhanced effects of the Toll and JAK-STAT pathways in shaping rural mosquito populations’ resistance to DENV. These findings align with previous studies demonstrating that Toll pathway transcription factors *Rel1* and *Rel2* induce the expression of antimicrobial peptides such as *DefA* and *CecA*, which exhibited potent antiviral properties [22, 23, 59, 73, 77, 78]. The consistent expression of *CecA* and *Rel2* across mosquito populations in our analysis underscores their conserved role in antiviral responses, suggesting their potential utility as biomarkers for targeted dengue vector control strategies. The role of *DefA* in immunity is particularly noteworthy, with its upregulation during viral infections contributing to effective pathogen defense. Inducing simultaneous expression of *DefA* and *CecA* in transgenic *Ae. aegypti* has been shown to eliminate parasitic infections [21, 22]. Similarly, *STAT* activation, which upregulates antiviral genes such as vago, restricts viral replication and plays a pivotal role in shaping mosquito antiviral defenses [73]. While much of the focus has been on DENV, the variable activation of these pathways in response to different arboviruses warrants further investigation. Future research should explore diverse approaches, including infection timing, genetic interference, and additional immune markers such as reactive oxygen species (ROS) and nitric oxide (NO), to uncover the complexities of mosquito immunity. Understanding the interplay between host factors, viral persistence, and species-specific permissiveness will provide valuable insights into arboviral transmission dynamics. Collectively, our findings highlight the sophisticated immune network of *Aedes* mosquitoes, underscoring its significance in VC and its potential as a target for innovative vector control strategies.

The findings of the study must be interpreted in light of several limitations. We did not conduct subsequent metagenomics analysis of the intestinal fauna among these mosquito populations, which could provide insights into potential factors influencing the observed differences in VC. For example, Zhang et al. [6] reported that *Rosenbergiella*_YN46, a commensal bacterium in *Ae. albopictus*, was linked to reduced flavivirus infections and a lower incidence of dengue in Yunnan Province, China. Furthermore, we did not investigate the potential influence of insect-specific viruses (ISVs), which naturally circulate in mosquito populations and may interfere with dengue VC, as ISVs have been shown to affect arbovirus replication in some mosquito species [79]. Future studies should consider these factors to provide a more comprehensive understanding of VC. Additionally, a phase-wise investigation of mosquito–virus–gut bacteria interactions could reveal phase-specific changes in the transmission cycle, enhancing our understanding of vector dynamics.

## Conclusions

This study explored the biological determinants that influence the ability of mosquito populations to transmit pathogens, with a particular emphasis on how these factors vary across different environmental settings. Mosquitoes from non-dengue areas (rural) exhibited reduced DENV RNA dissemination and transmission. Moreover, variation in VC may be associated with mosquito innate immune responses, involving *Rel1* and *DefA* in the Toll and Imd pathways, *STAT* in the JAK-STAT pathway, and the abundance of the *w*AlbB strain. The correlation analysis between immune gene expression and *Wolbachia* abundance suggested that complex regulatory networks govern host–pathogen interactions. This information is crucial for public health, as it helps identify priority areas for dengue surveillance and vector control. This emphasizes the need to consider the characteristics and behaviors of both urban and rural *Aedes* mosquitoes to effectively reduce virus transmission risk. However, we recommend more in-depth research on a sizable sample of mosquitoes from various environments to uncover additional plausible variables, such as the environment, genetics, and midgut microbiota, that could influence VC.

## Supplementary Information

Supplementary Material 1: Text S1. Primers used in this study.

Supplementary Material 2: Table 1. ANOVA and MLC tests for immune gene expression in different mosquito populations.

## Data Availability

No datasets were generated or analyzed during the current study.
