# Supplementary material for: Dynamics of vector competence for dengue virus type 2 in rural and urban populations of Aedes albopictus: implications for infectious disease control
Source: Parasit Vectors. 2025 Jun 1;18:201. doi: 10.1186/s13071-025-06826-8 (PMC12128243; doi:10.1186/s13071-025-06826-8)
Supplement: Supplementary file 1 — Supplementary Material 1: Text S1. Primers used in this study. [file 13071_2025_6826_MOESM1_ESM.docx]

1. **Cecropin A (CecA)**

FP: 5′-TGGCTGTTCTTCTCCTGACC-3′, RP 5′-CCCAGCTACAACAGGAAGA-3

1. **Defensin (DefA)**

FP: 5′-CCATGCAGCCCCTCACTGTCAT- 3′, RP: 5′- ATTCCGGCAGACGCACACCTT-3′

1. **Relish 1 (Rel1)**

FP: 5′-AGCATCGGCGAGATCAACAT-3′, RP: 5′-TCGAAACTGTCCGAGAGCGT-3′

1. **Rel2**

FP: 5′-AGTTTGAACGTTCTGCTGG-3′, RP: 5′-TTGTGTCCATGCTTCAGATC-3′

1. **The signal transducers and activators of transcription (STAT)**

FP: 5′-CACCGGATCGTTAACCCTG-3′, RP: 5′-AGCCATGGACACGTCGTC-3′

1. ***w*AlbA**

FP: 5′-GGGTTGATGTTGAAGGAG-3′, RP: 5′-CACCAGCTTTTACTTGACC-3′

1. ***w*AlbB**

FP: 5′-ACGTTGGTGGTGCAACATTTG-3′, RP: 5′-TAACGAGCACCAGCATAAAGC-3′

1. **Rps6**

FP: 5′-TACATGAAGATCCTGCACCTG-3′, RP 5′-CTTCTCCTTCTCCTTGTCGC-3′

1. **DENV**

FP: 5′-TCCCTTACAAATCGCAGCAAC-3′, RP: 5′-TGGTCTTTCCCAGCGTCAAT-3′
