# Supplementary material for: Dynamics of vector competence for dengue virus type 2 in rural and urban populations of Aedes albopictus: implications for infectious disease control
Source: Parasit Vectors. 2025 Jun 1;18:201. doi: 10.1186/s13071-025-06826-8 (PMC12128243; doi:10.1186/s13071-025-06826-8)
Supplement: Supplementary file 2 — Supplementary Material 2: Table 1. ANOVA and MLC tests for immune gene expression in different mosquito populations. [file 13071_2025_6826_MOESM2_ESM.docx]

**Table 1:** **ANOVA and MLC tests for immune genes expression in different mosquito populations**

| **Gene** | **Variable** | | | **Sum of Squares** | | **df** | | **Mean Square** | | **F** | | **Sig.** |
| --- | --- | --- | --- | --- | --- | --- | --- | --- | --- | --- | --- | --- |
| CecA | Between Groups | | | .321 | | 2 | | .160 | | .828 | | .448 |
|  | Within Groups | | | 5.229 | | 27 | | .194 | |  | |  |
|  | Total | | | 5.550 | | 29 | |  | |  | |  |
| Rel1 | Between Groups | | | 7.900 | | 2 | | 3.950 | | 7.119 | | .003 |
|  | Within Groups | | | 14.981 | | 27 | | .555 | |  | |  |
|  | Total | | | 22.882 | | 29 | |  | |  | |  |
| DefA | Between Groups | | | 6.112 | | 2 | | 3.056 | | 10.527 | | .000 |
|  | Within Groups | | | 7.839 | | 27 | | .290 | |  | |  |
|  | Total | | | 13.951 | | 29 | |  | |  | |  |
| Rel2 | Between Groups | | | .116 | | 2 | | .058 | | .113 | | .894 |
|  | Within Groups | | | 13.816 | | 27 | | .512 | |  | |  |
|  | Total | | | 13.932 | | 29 | |  | |  | |  |
| STAT | Between Groups | | | 2.831 | | 2 | | 1.416 | | 5.094 | | .013 |
|  | Within Groups | | | 7.503 | | 27 | | .278 | |  | |  |
|  | Total | | | 10.335 | | 29 | |  | |  | |  |
| **Multiple Comparisons test for immune genes expression in different mosquito populations** | | | | | | | | | | | | |
| Dependent Variable | | | Mean Difference (I-J) | | Std. Error | | Sig. | | 95% Confidence Interval | | | |
|  |  |  |  |  |  |  |  |  | Lower Bound | | Upper Bound | |
| Rel1 | lab | urban | -.07031 | | .33312 | | .834 | | -.7538 | | .6132 | |
|  |  | rural | -1.12206^*^ | | .33312 | | .002 | | -1.8056 | | -.4386 | |
|  | urban | rural | -1.05175^*^ | | .33312 | | .004 | | -1.7353 | | -.3682 | |
| DefA | lab | urban | .05093 | | .24097 | | .834 | | -.4435 | | .5454 | |
|  |  | rural | -.93105^*^ | | .24097 | | .001 | | -1.4255 | | -.4366 | |
|  | urban | rural | -.98198^*^ | | .24097 | | .000 | | -1.4764 | | -.4875 | |
| STAT | lab | urban | -.00588 | | .23576 | | .980 | | -.4896 | | .4779 | |
|  |  | rural | -.65462^*^ | | .23576 | | .010 | | -1.1383 | | -.1709 | |
|  | urban | rural | -.64874^*^ | | .23576 | | .010 | | -1.1325 | | -.1650 | |
| *. The mean difference is significant at the 0.05 level. | | | | | | | | | | | | |
